# A Meta-Analysis of the Association between Polymorphisms in MicroRNAs and Risk of Ischemic Stroke

**DOI:** 10.3390/genes6041283

**Published:** 2015-12-07

**Authors:** Yan Xiao, Mei-Hua Bao, Huai-Qing Luo, Ju Xiang, Jian-Ming Li

**Affiliations:** 1 Department of Anatomy, Histology and Embryology, Institute of Neuroscience, Changsha Medical University, Changsha 410219, China; E-Mails: luohuaiqing@163.com (H.-Q.L.); xiang.ju@foxmail.com (J.X.); 2 Qingdao Science & Standard Chemicals Analysing and Testing Co., Ltd., Qingdao 266000, China; E-Mail: y_xiao@sscta.cn

**Keywords:** miR-146a, miR-149, miR-196a2, miR-499, polymorphism, ischemic stroke, meta-analysis

## Abstract

Ischemic stroke (IS) is responsible for a high death rate and for adult disability worldwide. MiR-146a (rs2910164), miR-149 (rs2292832), miR-196a2 (rs11614913) and miR-499 (rs3746444) are found to be associated with ischemic stroke. However, the results were inconsistent and inconclusive. The present study performed a meta-analysis to get a more precise and comprehensive estimation of the association between the four polymorphisms and IS risk. The databases Pubmed, Embase, Cochrane Central Register of Controlled Trials, Chinese National Knowledge Infrastructure, and Chinese Biomedical Literature Database were searched for related studies. A total of five studies including 2230 cases and 2229 controls were identified for the meta-analysis. The results indicate that TT genotype and T allele of miR-149 (rs2292832) are associated with significantly lower risks of IS in a homozygous model (OR = 0.70) and an allelic model (OR = 0.86). No significant associations were found between miR-146a (rs2910164), miR-196a2 (rs11614913), miR-499 (3746444) and IS susceptibility in any of the studies. However, subgroup analysis by sample size indicates a significant decrease in risks of IS for CC genotype and C allele of miR-146a (rs2910164) in the large sample size group. Therefore, miR-149 (rs2292832) might be recommended as a predictor for IS risk, while miR-146a (rs2910164), miR-196a2 (rs11614913), miR-499 (3746444) are not.

## 1. Introduction

Being the most common kind of stroke, ischemic stroke (IS) is responsible for a high death rate and adult disability worldwide. The mortality rate was reported to be about 120–180 per 100,000 in China, and it has become the third leading cause of death in the USA, exceeding the death rate of heart diseases [1,2]. The risk factors of IS include age, smoking, diabetes mellitus, and hyperlipemia. Additionally, the genetic mutants were found to play important roles in IS susceptibility [3,4].

MicroRNAs are non-coding, small RNAs with lengths of about 22 nt, which regulate the expression of many genes at a post-transcriptional level by binding to the 3′-UTR of target genes [5,6]. A slight change in the sequence of miRNAs, such as single nucleotide polymorphisms (SNPs), may change their production or affinity to target genes and result in diseases. Recently, polymorphisms in pre-miRNA sequences of miR-146a C>G (rs2910164), miR-149 T>C (rs2292832), miR-196a2 T>C (rs11614913) and miR-499 A>G (rs3746444) have been found [7,8]. According to recent data, the miR-146aG, miR-149T, miR-196a2C, and miR-499G alleles are possible genetic predisposing factors, and may associate with ischemic stroke. However, the results were inconsistent and inconclusive due to the sample size, ethnicity or source of control, *etc*. Thus, in the present study, we included a total of 5 studies, which have 2230 cases and 2229 controls, to get a more precise and comprehensive estimation of the association between the four polymorphisms and IS.

## 2. Experimental Section

### 2.1. Publication Search Strategy and Inclusion Criteria

We systematically searched the published studies in the electronic databases of Pubmed, Embase, Cochrane Central Register of Controlled Trials (CENTRAL), Chinese National Knowledge Infrastructure (CNKI) and Chinese Biomedical Literature Database (CBM). The searched terms are as follows: (“Ischemic stroke”) and (“microRNA”) and (“polymorphism” or “mutation” or “variant” or “SNP” or “single nucleotide polymorphism”). There were no restrictions on language. The deadline for publication was 24 August 2015. All the results from the databases were screened. First, we screened the titles. If a title fulfilled our criteria, we then screened the abstract. We retrieved the full text if the abstract was in accordance with our interest. All eligible studies were retrieved manually for other potentially relevant studies from their references. We would have contacted the authors for related data if they were unavailable in the original publications.

Inclusion Criteria: (1) a case-control design; (2) that the association between microRNA polymorphisms and IS risks has been evaluated; (3) that the genotype in the control group is in agreement with the Hardy-Weinberg equilibrium (HWE) [9]; and (4) that the data in the publication are sufficient for present estimation. Studies were excluded if any of the following applied: (1) repeated publications, abstracts, letters or reviews; and (2) studies that do not meet all of the inclusion criteria.

### 2.2. Data Extraction

Information was extracted from each eligible publication manually by two investigators independently (Y. Xiao and J. M. Li). For each study, the extracted information included first authors’ name, published year, country, ethnicity, genotype method, the source of control, sex and age match, and the genotype numbers of cases and controls. If there were discrepancies during data extraction, it would be resolved by a consensus achieved by a third author (M. H. Bao).

### 2.3. Quality Assessment

The quality of included studies was evaluated according to the predefined scale for quality assessment provided by Jiang *et al.* [10]. The score scale includes the following aspects: the source of cases (those selected from population or cancer registry were given a score of 3; those selected from hospital, 2; those selected from pathology archives but without description, 1; and 0 for other cases), the source of controls (population-based controls, 3; blood donors or volunteers, 2; hospital-based cancer-free patients, 1; and others, 0), specimens used for determining genotypes (white blood cells or normal tissues, 3; and tumor tissues or exfoliated cells of tissue, 0), total sample size (>1000 was scored 3; >500 and <1000, 2; >200 and <500, 1; and <200, 0) and evidence of HWE (in HWE, 3, and out of HWE, 0). The quality scores ranged from 0–15. Reports with scores <10 were classified as “low quality”, and those with scores ≥10 were regarded as “high quality”. The quality evaluation was performed by two authors independently (M.H. Bao and J. Xiang). Consensus achieved by a third author was reached to resolve any discrepancies in the assessment process.

### 2.4. Statistical Methods

The χ^2^-test was used to evaluate the HWE of the control group polymorphism. If *p* < 0.05, it is considered to be deviated from HWE. To evaluate the association between microRNA polymorphisms and IS risk, the crude odds ratio (OR) with 95% confidence interval (CI) was used. The summary effect estimate OR was calculated using genetic models of homozygous, heterozygous, dominant and allelic model. The statistical significance was determined by the *Z*-test, and *p* < 0.05 was considered to be statistically significant. Subgroup analysis by sample size was conducted. Groups with total samples less than 1000 was treated as small or as large.

The Q-statistic was used to test for homogeneity, and the *I*^2^ and 95% *I*^2^ was used to assess the degree of heterogeneity [11]. If there is an obvious heterogeneity (*I*^2^ > 50%) or a moderate heterogeneity (*I*^2^ 25%–50%) among the studies, the random-effects model (the DerSimonian and Laird method) was used for the meta-analysis [12]. Otherwise, the fixed-effect model using the Mantel-Haenszel method was used [13]. Sensitivity analysis was performed to assess the effects of individual study on the summarized effects estimate and the stability of results. The publication bias was detected with Begg’s funnel plot and Egger’s linear regression method, and *p* < 0.05 was considered to be statistically significant [14]. All statistical analysis was performed using the STATA 12.0 software (Stata Corp., College Station, TX, USA) and Revman 5.3.

## 3. Results and Discussion

### 3.1. Characteristics of Eligible Studies

**Figure 1:**
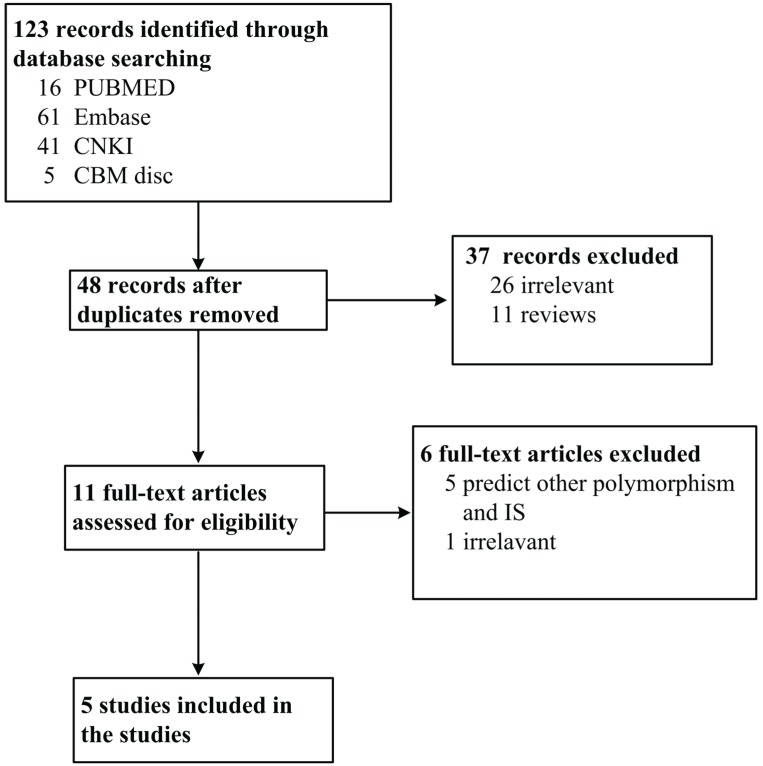
PRISMA flow chart of studies inclusion and exclusion.

A total of 48 studies were obtained from the literature search after duplicates removed. Among them, 27 studies were excluded for irrelevance, 11 for being reviews, 5 for predicting other polymorphisms and IS. At last, five studies were evaluated as meeting the criteria, which included 2230 cases and 2229 controls. Among these studies, four are on the association between miR-146a rs2910164 and IS, two on the association between miR-149 rs2292832 and IS, four on the association between miR-196a2 rs11614913 and IS, and three on the association between miR-499 rs3746444 and IS. The PRISMA flow chart is shown in Figure 1, and the information for included studies is presented in Table 1.

**Table 1:** Characteristics of eligible studies included in the meta-analysis.

**miR-146a rs2910164**													
**Author**	**Year**	**Country**	**Ethnicity**	**Genotyping Methods**	**Sex Ratio (Male:Female) (Case/Control)**	**Age (Case/Control)**	**Quality Score**	**Sample Size (Case/Control)**	**GG**	**GC**	**CC**	**HWE of Control**	**Source of Control**
									**(Case/Control)**	**(Case/Control)**	**(Case/Control)**		
Zhu [15]	2014	China	Chinese	PCR-LDR	253:115/261:120	61.62 ± 0.986/62.05 ± 0.982	12	368/381	50/64	173/185	145/132	0.384	volunteers
Liu [16]	2014	China	Chinese	PCR-RFLP	180:116/127:193	67.52 ± 10.29/66.34 ± 11.07	12	296/391	52/77	159/198	85/116	0.650	volunteers
Jeon [4]	2013	South Korea	Korean	Taqman	336:342/244:309	64.16 ± 11.90/63.14 ± 10.19	12	678/553	128/76	327/266	223/211	0.589	IS-free patients
Huang [17]	2015	China	Chinese	Taqman	327:204/327:204	63 (54, 70)/61 (54, 68)	13	531/531	81/55	261/257	189/219	0.106	volunteers
**miR-149rs2292832**													
**Author**	**Year**	**Country**	**Ethnicity**	**Genotyping Methods**	**Sex Ratio (Male:Female) (Case/Control)**	**Age (Case/Control)**	**Quality Score**	**Sample Size (Case/Control)**	**TT**	**TC**	**CC**	**HWE of Control**	**Source of Control**
									**(Case/Control)**	**(Case/Control)**	**(Case/Control)**		
Jeon [4]	2013	South Korea	Korean	Taqman	336:342/244:309	64.16 ± 11.90/63.14 ± 10.19	12	678/553	299/262	303/238	76/53	0.589	IS-free patients
He [18]	2013	China	Chinese	PCR-RFLP	205:168/193:180	65.7 ± 11.5/66.3 ± 10.2	12	357/373	138/160	162/175	57/38	0.327	volunteers
**miR-196a2rs11614913**													
**Author**	**Year**	**Country**	**Ethnicity**	**Genotyping Methods**	**Sex Ratio (Male:Female) (Case/Control)**	**Age (Case/Control)**	**Quality Score**	**Sample Size (Case/Control)**	**CC**	**CT**	**TT**	**HWE of Control**	**Source of Control**
									**(Case/Control)**	**(Case/Control)**	**(Case/Control)**		
Zhu [15]	2014	China	Chinese	PCR-LDR	253:115/261:120	61.62 ± 0.986/62.05 ± 0.982	12	368/381	71/78	189/198	108/105	0.384	volunteers
Liu [16]	2014	China	Chinese	PCR-RFLP	180:116/127:193	67.52 ± 10.29/66.34 ± 11.07	12	296/391	51/84	181/214	64/93	0.650	volunteers
Jeon [4]	2013	South Korea	Korean	Taqman	336:342/244:309	64.16 ± 11.90/63.14 ± 10.19	12	678/553	139/105	352/292	187/156	0.589	IS-free patients
Huang [17]	2015	China	Chinese	Taqman	327:204/327:204	63 (54, 70)/61 (54, 68)	13	531/531	100/112	265/266	166/153	0.106	volunteers
**MiR-499 rs3746444**													
**Author**	**Year**	**Country**	**Ethnicity**	**Genotyping Methods**	**Sex Ratio (Male:Female) (Case/Control)**	**Age (Case/Control)**	**Quality Score**	**Sample Size (Case/Control)**	**AA**	**AG**	**GG**	**HWE of Control**	**Source of Control**
									**(Case/Control)**	**(Case/Control)**	**(Case/Control)**		
Liu [16]	2014	China	Chinese	PCR-RFLP	180:116/127:193	67.52 ± 10.29/66.34 ± 11.07	12	296/391	181/278	96/99	19/14	0.650	volunteers
Jeon [4]	2013	South Korea	Korean	Taqman	336:342/244:309	64.16 ± 11.90/63.14 ± 10.19	12	678/553	460/365	195/170	23/18	0.589	IS-free patients
Huang [18]	2015	China	Chinese	Taqman	327:204/327:204	63 (54, 70)/61 (54, 68)	13	531/531	398/403	133/128	0/0	0.106	volunteers

HWE: Hardy-Weinberg equilibrium; PCR-RFLP: polymerase chain reaction-Restriction fragment length polymorphism; PCR-LDR: polymerase chain reaction-ligation detection reaction. The HWE of the control group polymorphism was evaluated by the χ^2^-test.

### 3.2. Results of Meta-Analysis

**Figure 2:**
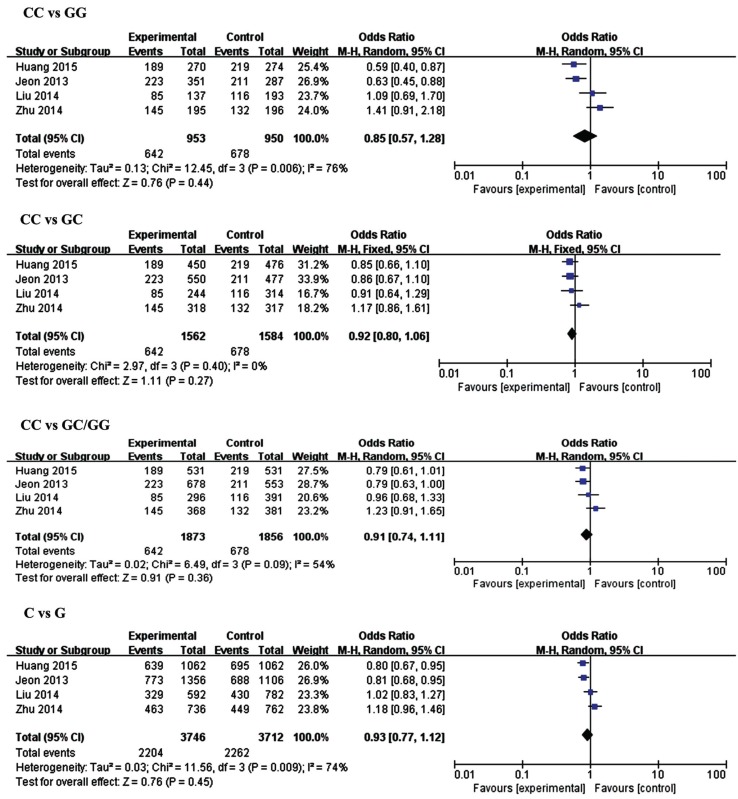
Forest plots of the odds ratio for the association between microRNA-146a rs2910164 and risks of IS. The summary effect estimate was detected by the *Z*-test. *p* < 0.05 was considered to be statistically significant. The Q-statistic was used to test for homogeneity, and the *I*^2^ was used to assess the degree of heterogeneity. The random-effects model was used for the meta-analysis when *I*^2^ > 25%. Otherwise, the fixed-effect model was used.

**Figure 3:**
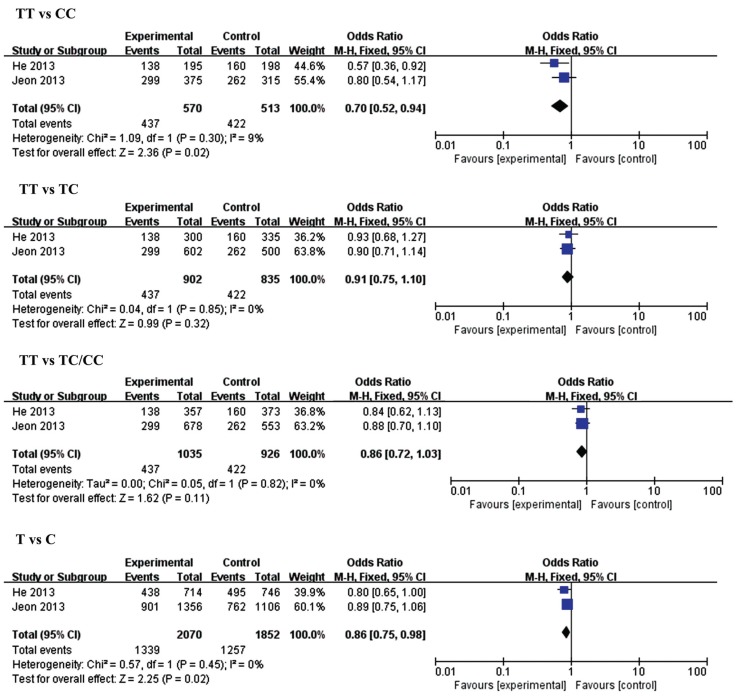
Forest plots of the odds ratio for the association between microRNA-149 rs2292832 and risks of IS. The summary effect estimate was detected by the *Z*-test. *p* < 0.05 was considered to be statistically significant. The Q-statistic was used to test for homogeneity, and the *I*^2^ was used to assess the degree of heterogeneity. The random-effects model was used for the meta-analysis when *I*^2^ > 25%. Otherwise, the fixed-effect model was used.

**Figure 4:**
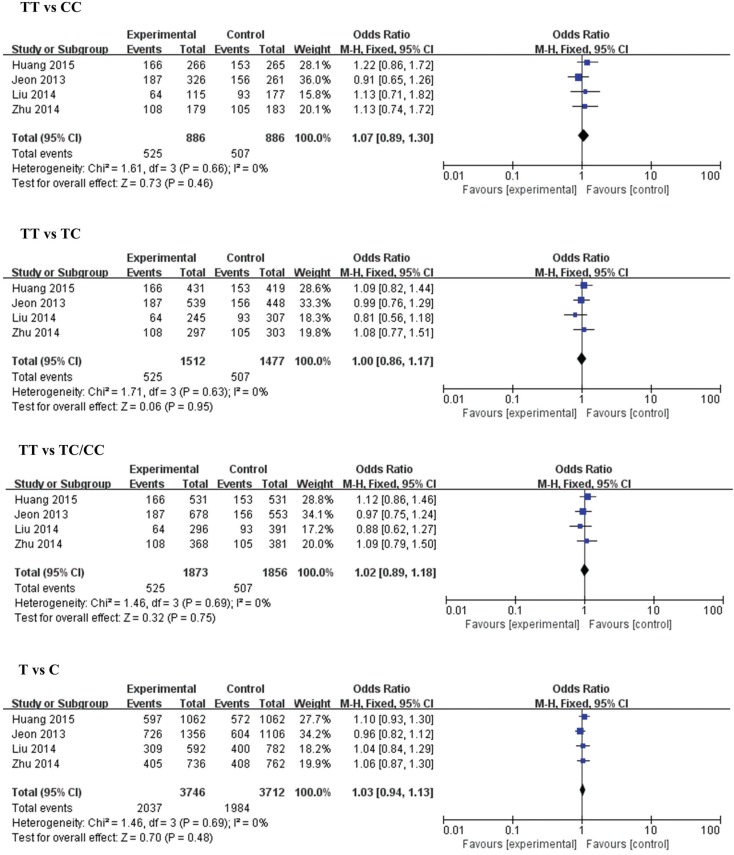
Forest plots of the odds ratio for the association between microRNA-196a2 rs711614913 and risks of IS. The summary effect estimate was detected by the *Z*-test. *p* < 0.05 was considered to be statistically significant. The Q-statistic was used to test for homogeneity, and the *I*^2^ was used to assess the degree of heterogeneity. The random-effects model was used for the meta-analysis when *I*^2^ > 25%. Otherwise, the fixed-effect model was used.

**Figure 5:**
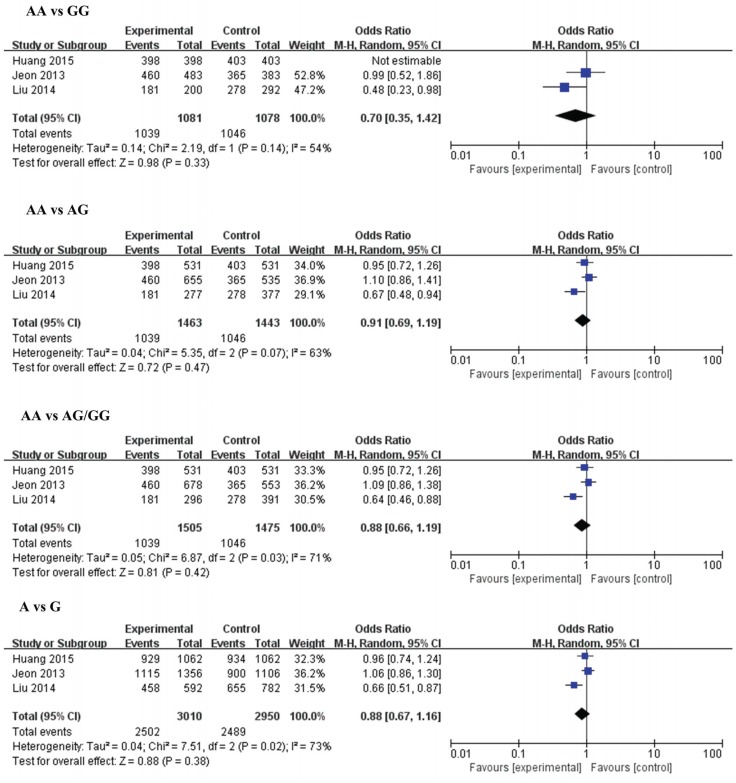
Forest plots of the odds ratio for the association between microRNA-499 rs3746444 and risks of IS. The summary effect estimate was detected by the *Z*-test. *p* < 0.05 was considered to be statistically significant. The Q-statistic was used to test for homogeneity, and the *I*^2^ was used to assess the degree of heterogeneity. The random-effects model was used for the meta-analysis when *I*^2^ > 25%. Otherwise, the fixed-effect model was used.

The results of meta-analysis for the association between miR-146a (rs2910164), miR-149 (rs2292832), miR-196a2 (rs11614913), miR-499 (rs3746444) and ischemic stroke risks are shown in Table 2 and Figure 2, Figure 3, Figure 4 and Figure 5.

**Table 2:** Summary effect estimate ORs and 95% CIs of the association between miR-146a (rs2910164), miR-149 (rs2292832), miR-196a2 (rs11614913), miR-499 (rs3746444) and ischemic stroke (IS).

**Genetic Model**		**MiR-146a rs2910264**				**Genetic Model**		**MiR-149 rs2292832**			
		***N***	**OR (95% CI)**	***p*-Value**	***I*^2^ (%)**			***N***	**OR (95% CI)**	***p*-Value**	***I*^2^ (%)**
CC *vs.* GG	Overall	4	0.85 (0.57,1.28)	0.44	76	TT *vs.* CC	Overall	2	0.70 (0.52, 0.94)	0.02	9
	Small size	2	1.24 (0.91, 1.70)	0.18	0		Small size	1	0.57 (0.36, 0.92)	0.02	N.A
	Large size	2	0.61 (0.47, 0.79)	0.0002	0		Large size	1	0.80 (0.54, 1.17)	0.25	N.A
CC *vs.* GC	Overall	4	0.92 (0.80,1.06)	0.27	0	TT *vs.* TC	Overall	2	0.91 (0.75, 1.10)	0.32	0
	Small size	2	1.05 (0.83, 1.32)	0.69	10		Small size	1	0.93 (0.68, 1.27)	0.66	N.A
	Large size	2	0.85 (0.71, 1.02)	0.09	0		Large size	1	0.90 (0.71, 1.14)	0.37	N.A
CC *vs.* GC/CC	Overall	4	0.89 (0.78, 1.02)	0.10	54	TT *vs.* TC/CC	Overall	2	0.86 (0.72, 1.03)	0.11	0
	Small size	2	1.10 (0.88, 1.37)	0.41	17		Small size	1	0.84 (0.62, 1.13)	0.24	81
	Large size	2	0.79 (0.67, 0.94)	0.007	0		Large size	1	0.88 (0.70, 1.10)	0.25	N.A
C *vs.* G	Overall	4	0.93 (0.77, 1.12)	0.45	74	T *vs.* C	Overall	2	0.86 (0.75, 0.98)	0.02	0
	Small size	2	1.10 (0.95, 1.28)	0.20	0		Small size	1	0.80 (0.65, 1.00)	0.05	N.A
	Large size	2	0.80 [0.71, 0.90)	0.0003	0		Large size	1	0.89 [0.75, 1.06)	0.20	N.A
**Genetic Model**		**MiR-196a2 rs 11614913**				**Genetic Model**		**MiR-499 rs3746444**			
		***N***	**OR (95% CI)**	***p*-Value**	***I^2^* (%)**			***N***	**OR (95% CI)**	***p*-Value**	***I^2^* (%)**
TT *vs.* CC	Overall	4	1.07 [0.89, 1.30)	0.46	0	AA *vs.* GG	Overall	3	0.70 [0.35, 1.42)	0.33	54
	Small size	2	1.13 [0.83, 1.55)	0.44	0		Small size	1	0.48 [0.23, 0.98)	0.04	N.A
	Large size	2	1.04 [0.78, 1.39)	0.77	31		Large size	2	0.99 [0.52, 1.86)	0.97	N.A
TT *vs.* TC	Overall	4	1.00 [0.86, 1.17)	0.95	0	AA *vs.* AG	Overall	3	0.87 [0.54, 1.41)	0.57	81
	Small size	2	0.95 [0.74, 1.22)	0.69	17		Small size	1	0.67 [0.48, 0.94)	0.02	N.A
	Large size	2	1.04 [0.86, 1.26)	0.70	0		Large size	2	1.03 [0.86, 1.24)	0.75	0
TT *vs.* TC/CC	Overall	4	1.02 (0.89, 1.18)	0.75	0	AA *vs.* AG/GG	Overall	3	0.84 (0.50, 1.42)	0.52	85
	Small size	2	1.00 (0.78, 1.26)	0.97	0		Small size	1	0.64 (0.46, 0.88)	0.006	N.A
	Large size	2	1.04 (0.87, 1.25)	0.67	0		Large size	2	1.03 (0.86, 1.23)	0.77	0
T *vs.* C	Overall	4	1.03 (0.94, 1.13)	0.48	0	A *vs.* G	Overall	3	0.84 (0.53, 1.34)	0.47	86
	Small size	2	1.05 (0.91, 1.22)	0.50	0		Small size	1	0.66 (0.51, 0.87)	0.003	N.A
	Large size	2	1.02 (0.89, 1.17)	0.75	26		Large size	2	1.02 (0.87, 1.20)	0.82	0

The summary effect estimate ORs and CIs was detected by the *Z*-test. *p* < 0.05 was considered to be statistically significant. Groups with total samples less than 1000 were treated as small or as large. The Q-statistic was used to test for homogeneity, and the *I*^2^ was used to assess the degree of heterogeneity. When *I*^2^ > 50%, the random-effects model (the DerSimonian and Laird method) was used for the meta-analysis. Otherwise, the fixed-effect model using the Mantel-Haenszel method was used. N.A: not applicable.

#### 3.2.1. MiR-146a (rs2910164) and IS

Four studies with 1873 cases and 1856 controls were included in this analysis. No significant associations were found between rs2910164 and IS susceptibility in the overall analysis for all genetic models. However, subgroup analysis by sample size showed a significant decrease in risks of IS in large sample size groups in the homozygous model (CC *vs.* GG, OR = 0.61, 95% CI = 0.47–0.79, *p* = 0.0002), the dominant model (CC *vs.* GC/GG, OR = 0.79, 95% CI = 0.67–0.94, *p* = 0.007) and the allelic model (C *vs.* G, OR = 0.80, 95% CI = 0.71–0.90, *p* = 0.0003).

#### 3.2.2. MiR-149 (rs2292832) and IS

Two studies with 1035 cases and 926 controls were included in this analysis. A significant decrease in ischemic stroke risks was found in the homozygous model (TT *vs.* CC, OR = 0.70, 95% CI = 0.52–0.94, *p* = 0.02) and the allelic model (T *vs.* C, OR = 0.86, 95% CI = 0.75–0.98, *p* = 0.02). The same relation was found in the small sample size group.

#### 3.2.3. MiR-196a2 (rs11614913) and IS

Four studies with 1873 cases and 1856 controls were included in this analysis. No significant associations were found in the overall analysis or subgroup analysis in any of the models, be they homozygous, heterozygous, dominant or allelic. Since a moderate heterogeneity was found in the large group of homozygous models (TT *vs.* CC) and allelic models (T *vs.* C), we also conducted a meta-analysis using the fixed effects model. No significant difference between these two models were found (TT *vs.* CC, fixed model: OR = 1.04, 95% CI = 0.82–1.32, *p* = 0.74; random model: OR = 1.04, 95% CI = 0.78–1.39, *p* = 0.77. T *vs.* C, fixed model: OR = 1.02, 95% CI = 0.91–1.15, *p* = 0.72; random model: OR = 1.02, 95% CI = 0.89–1.17, *p* = 0.75).

#### 3.2.4. MiR-499 (rs3746444) and IS

Three studies with 1505 cases and 1476 controls were included in this analysis. No significant associations were found between rs3746444 and IS susceptibility in the overall analysis. In subgroup analysis, no associations were found in large sample size groups, while a significantly decreased risk of IS was found in the small sample size group.

### 3.3. Sources of Heterogeneity

Since significant heterogeneity was found for miR-146 (rs2910164) and miR-499 (rs3746444), we performed a subgroup analysis by sample size to explore the source of this heterogeneity. The results indicate that the sample size is the source of heterogeneity for miR-146 (rs2910164) and miR-499 (rs3746444) in every genetic model.

### 3.4. Sensitivity Analysis

The influence of each study on the summary effect estimate ORs and 95% CIs were evaluated by excluding one single study at a time using STATA 12.0 software. No significant altered summary effect estimate ORs were found in any of the genetic models for miR-146a (rs2910164), miR-149 (rs2292832), miR-196a2 (rs11614913) and miR-499 (rs3746444).

### 3.5. Publication Bias

We performed the Begg’s funnel plot and Egger’s test to evaluate the publication bias. The *p* values for Egger’s linear regression tests are shown in Table 3. As the results indicated, no obvious publication bias was observed. Furthermore, these results were also demonstrated by the shape of the funnel plot.

**Table 3:** Egger’s linear regression test for funnel plot asymmetries.

	Genetic Models	*p* Value		Genetic Models	*p* Value
MiR-146	CC *vs.* GG	0.191	MiR-149	TT *vs.* CC	0.918
	CC *vs.* GC	0.420		TT *vs.* TC	0.547
	CC *vs.* GC/CC	0.276		TT *vs.* TC/CC	0.684
	C *vs.* G	0.110		T *vs.* C	0.758
MiR-196a2	TT *vs.* CC	0.569	MiR-499	AA *vs.* GG	Not estimated
	TT *vs.* TC	0.413		AA *vs.* AG	0.392
	TT *vs.* TC/CC	0.630		AA *vs.* AG/GG	0.185
	T *vs.* C	0.563		A *vs.* G	0.405

## 4. Discussion

The main findings of the present meta-analysis are that the TT genotype and the T allele of miR-149 (rs2292832) are associated with significantly lower risks of IS in the homozygous model (OR = 0.70) and the allelic model (OR = 0.86). There were no significant associations found between miR-146a (rs2910164), miR-196a2 (rs11614913), miR-499 (3746444) and IS susceptibility in any of the studies. However, subgroup analysis by sample size indicates a significant decrease in risks of IS for the CC genotype and the C allele of miR-146a (rs2910164) in the large sample size group.

Significant heterogeneity was found in the meta-analysis for miR-146a (rs2910164) and miR-499 (rs3746444). Thus, we conducted a subgroup analysis by sample size and observed that sample size was the source of the heterogeneity. However, only one study was included in the small sample size subgroup of miR-499, and two studies were included in the large sample size group of miR-499. For miR-146a, there were only two studies in either of the small and large sample size groups. Thus, further studies are needed to confirm these results. In the sensitivity analysis, no significant changes were found after omitting one study at a time, indicating the relative stability and credibility of the results of our meta-analysis.

Previous studies have demonstrated that the mutation in pre-miRNA of miR-146a, miR-149, miR-196a2 and miR-499 decrease the mature miRNA levels [17,19,20]. These four microRNAs affect thrombosisor inflammation pathways in the circulation system by regulating tumor necrosisfactor-α(TNF-α) [21], methylenetetrahydrofolate reductase (MTHFR) [22], annexin A1 (ANXA1) [23], C-reactive protein (CRP) [24] and NF-κB pathway as well as the MAP kinase pathway [25]. Since these molecules all participate in the pathogenesis of IS, the four polymorphisms might contribute to the pathogenesis of IS. However, we only found the relation between miR-149 (rs2292832) and IS in the present meta-analysis. A previous study has demonstrated that MTHFR is the target of miR-149. MTHFR is a key enzyme in the methionine-folacin metabolism pathway. It is found to be associated with the risk of ischemic stroke [26]. The SNP in the pre-miR-149 changes the T allele to C, and, as a result, influences the mature miR-149 production. The change of mature miR-149 production then subsequently influences the folate metabolism pathway by targeting MTHFR. However, only two studies were included in the present meta-analysis for miR-149 rs2292832. Further studies are still needed to confirm the associations.

Our meta-analysis did not find significant associations between miR-146a (rs2910164), miR-196a2 (rs11614913), miR-499 (rs3746444) and IS susceptibility in any of the studies. However, in the subgroup analysis, the CC genotype or C allele of miR-146a (rs2910164) were related to, and decrease the risk of, IS in the large sample size group as we previously reported [27]. MiR-146 is shown to regulate the NF-κB-induced inflammatory process and the Toll-like receptor (TLR) system [28]. The SNP in the pre-miR-146a changes the G allele to C, and thus influences the mature miR-146a production. The change of mature miR-146a production then subsequently influence the inflammatory process of IS. However, since we did not find association between rs2910164 and IS in overall samples, further evaluation on the results are still needed.

Our meta-analysis did not find associations between miR-196a2 (rs11614913), miR-499 (rs3746444) and IS. However, these two SNPs have been demonstrated to be related to other human diseases, including congenital heart disease [29], coronary heart disease [30] and cancer [31], or related to IS when combined with other SNPs [4]. Therefore, the effects of these polymorphisms might be dependent on pathological differences of human diseases in specific organs.

The results of the present meta-analysis should be interpreted carefully because of the following limitations. Firstly, the number of patients was relatively small, which may influence the outcomes. After a very comprehensive literature search from several different databases, only a total of five studies were included in the present meta-analysis. Among the studies, four are on the associations between miR-146a rs2910164 and IS (1873 cases and 1856 controls), two on the association between miR-149 rs2292832 and IS (1035 cases and 926 controls), four on the association between miR-196a2 rs11614913 and IS (1035 cases and 926 controls), and three on the association between miR-499 rs3746444 and IS (1505 cases and 1476 controls).

The second limitation is that the clinicpathological characteristics or disease subtypes are limited in most of these studies. Thirdly, ischemic stroke is a multi-factorial disease influenced by both genetic and environmental factors. The gene-gene and gene-environment interactions may play important roles in the functions of these four polymorphisms, but most studies lack the information about environmental exposure and multiple SNPs in miRNA-encoding genes. The fourth limitation lies in the ethnicity of the subjects. All the patients in the present study were Asians, and this limited the general application of the results to other populations.

## 5. Conclusions

In conclusion, the current meta-analysis suggests a decreased risk of IS for the TT and T allele of miR-149 (rs2292832), while miR-146a (rs2910164), miR-196a2 (rs11614913), miR-499 (3746444) are not associated with the risk of IS. Thus, miR-149 (rs2292832) might be recommended as a predictor for susceptibility of IS. However, the results of this meta-analysis should be interpreted with caution because of the heterogeneity among study designs. Further studies are needed to evaluate the associations between rs2910164 and these two diseases, especially in a large sample size, in Caucasians, and with clinic-pathological characteristics.
